# Competition, standards, and EU data spaces

**DOI:** 10.1016/j.dib.2025.111964

**Published:** 2025-08-07

**Authors:** M. Konrad Borowicz

**Affiliations:** Tilburg Institute for Law, Technology and Society, Tilburg Law School, Tilburg University, Tilburg, Netherlands

## Abstract

The 2023 Proposal for a Financial Data Access Regulation (FIDA) aims to significantly enhance competition in the financial services sector by extending data portability rights far beyond the scope initially established by the Second Payment Services Directive (PSD2). Under FIDA, consumers gain extensive rights to authorize third-party access to previously siloed financial data, thereby promoting competition among incumbent institutions and new entrants. Yet, FIDA’s ambition hinges on structured collaboration among competitors—raising significant competition law concerns under Article 101 TFEU. FIDA explicitly recognizes this risk. Its governance framework, particularly Article 10, clearly incorporates key principles of EU competition law, closely aligning financial data sharing schemes with the European Commission’s Guidelines on the applicability of Article 101 to horizontal cooperation agreements. However, FIDA does not directly regulate or prescribe the substance of the standards themselves. This raises an important question: can a governance framework alone, without mandatory substantive standards, effectively prevent competition risks in the schemes? This article argues that the absence of prescribed substantive standards under FIDA could lead to competition issues similar to those observed under PSD2, where fragmented technical interfaces caused interoperability failures, insufficient incentives, discouraged investment in data-sharing infrastructure, and market trust declined, thereby limiting consumer uptake. This risk holds implications beyond financial services, serving as an important reference point for the broader EU agenda on sectoral data spaces and open data initiatives, where similar governance challenges and competition risks are likely to emerge.

## 1 Introduction

The creation of financial data sharing schemes (hereinafter, the “schemes”) envisaged under the 2023 Proposal for a Financial Data Access Regulation (FIDA) marks a pivotal shift in the European Commission’s vision for a digital single market for financial services articulated in the 2020 Digital Finance Strategy (hereinafter, the “Strategy”). A central pillar of the Strategy is financial data “portability” or the right of consumers to request that data holders (typically, incumbent financial institutions) share it with data users (typically, challenger providers of financial services). What began in 2015 with the Second Payment Service Directive’s (PSD2) tentative foray into portability of payment accounts data, under the Commission’s Strategy broadens dramatically: FIDA extends the vision of portability to encompass a vast new landscape of consumer financial data—about mortgage agreements, loans, pensions, investments, and beyond. With this expansion comes an ambitious promise: that structured collaboration between market actors can unleash innovation, empower consumers, and drive competition in financial services where it has been lacking [1].

Consider, for instance, a fintech startup seeking to offer personalized investment advice to European consumers. Under FIDA, this startup could rely on the schemes to access standardized data on users’ savings accounts, pension contributions, and investment holdings—data previously siloed within large incumbent banks, pension and investment funds, respectively. In practice, however, the startup might face prohibitively high access fees, opaque data formats, or technical standards designed to favor legacy systems—barriers that could arise under a poorly structured scheme.

The promise of these schemes—and of the Commission’s broader Strategy for Data, which has recently moved from horizontal regulations like the Data Act, Data Governance Act, and Digital Markets Act towards sector-specific “data spaces”—is therefore paradoxical. Legally, the schemes are expected to take the form of a “collective contractual agreement between data holders and data users”—a phrase, that, to anyone remotely familiar with competition law, might resonate less like innovation and more like collusion. By encouraging coordination among competitors, the schemes raise foundational concerns under Article 101 of the Treaty on the Functioning of the European Union (TFEU). If not carefully designed, they risk entrenching incumbents, excluding challengers, and turning access fees and standards into subtle instruments of market foreclosure.

FIDA explicitly recognizes this risk. Its governance framework, particularly Article 10, clearly incorporates key principles of EU competition law, closely aligning financial data sharing schemes with the European Commission’s Guidelines on the applicability of Article 101 to horizontal cooperation agreements (hereinafter, the “Guidelines”), especially the section focused on technical standard-setting. This is unsurprising, given that a core purpose of FIDA schemes is precisely to establish common standards for data formats and technical interfaces, enabling customer-authorized data sharing.

Consistent with the Guidelines and the practice of standardization [2], FIDA emphasizes the governance and decision-making processes underpinning these standard-setting arrangements, aiming to ensure pro-competitive outcomes. However, it does not directly regulate or prescribe the substance of the standards themselves. This raises an important question: can a governance framework alone, without mandatory substantive standards, effectively prevent competition risks in the schemes? This article argues that the absence of prescribed substantive standards under FIDA could lead to competition issues similar to those observed under PSD2, where fragmented technical interfaces caused interoperability failures, insufficient incentives discouraged investment in data-sharing infrastructure, and market trust declined, thereby limiting consumer uptake [3, p. 77]. FIDA risks repeating this outcome although the Commission’s backstop authority to intervene directly after 18 months creates a strong incentive for the industry to accelerate efforts toward implementing the framework.

The issues raised by FIDA intersect with broader academic debates on data governance and competition. The debates have previously concentrated on corporate platforms [4], scholars increasingly emphasize alternative governance structures, such as cooperatives, trusts, or data pools [5]. For instance, Lundqvist examines the economic and legal implications of data pools, defined as centralized repositories managed collectively by firms to leverage analytics and artificial intelligence in sectors such as autonomous vehicles or personalized medicine [6]. While Lundqvist recognizes the innovation potential of these arrangements, he also highlights their significant competition risks, including facilitating collusion, exclusion, and abuses of market power. This article shares Lundqvist’s caution regarding the competitive dangers of unchecked collaborative data infrastructures.

However, the schemes introduced under FIDA differ fundamentally from traditional data pools. Rather than aggregating data centrally, FIDA schemes facilitate decentralized, bilateral data exchanges between individual data holders and data users, contingent on consumer authorization and governed by collectively established but not centrally enforced standards [7]. The Commission’s terminology—describing the resulting ecosystem as a “data space”—can thus be misleading, as it implies centralization akin to pooling rather than decentralized exchanges. Thus, although Lundqvist’s analysis of data pools offers valuable insights, the specific economic and competitive characteristics of FIDA schemes necessitate a distinct analytical framework under Article 101(3).

The analysis proceeds in three parts. First, it evaluates the specific features of the FIDA schemes that may restrict competition—either by object or effect. Second, it considers whether they qualify for exemption under Article 101(3). Third, it turns beyond legal formality to institutional reality, examining how the absence of mandatory substantive standards under FIDA could replicate past interoperability failures of PSD2, weaken investment incentives, and ultimately hinder innovation.

## 2 How is Competition Law Shaping the Governance Design of the Schemes?

The schemes are inherently collaborative agreements, involving data holders, data users and consumers. Under FIDA, the parties to these agreements are expected to develop common standards for the data and the technical interfaces to allow customers to request data sharing. For example, a consortium of banks and fintech companies might jointly agree on a common specification for application programing interfaces (APIs) that enables secure transmission of investment portfolio data. The agreement could include standardized formats for portfolio holdings, authentication protocols for consumer consent, and a tiered pricing model for data access based on usage volume. While such coordination facilitates interoperability and innovation, it also requires scrutiny under competition law to ensure it does not restrict access or foreclose rivals.

Specifically, such schemes must be analyzed under Article 101 TFEU, which governs agreements between undertakings. Accordingly, this Section of the article begins by identifying the potentially restrictive elements of the schemes. In identifying the potentially restrictive elements, it distinguishes between restrictions by object, which are inherently anti-competitive, and restrictions by effect, which require an assessment of their actual or potential impact on competition.

## 3 Do the Schemes Restrict Competition by Object?

A restriction by object refers to agreements that are inherently harmful to competition, with no need to analyze their actual effects. These agreements have such obvious anti-competitive purposes that their mere existence is deemed unlawful under Article 101(1) TFEU. In the context of the schemes, restrictions by object arise when scheme members explicitly agree on prices for data access.

An agreement on prices or price fixing are the quintessential example of an agreement that restricts competition [8]. Pursuant to such an agreement, competitors seek to coordinate their pricing strategies rather than allowing prices to be determined independently by market forces. Such coordination is inherently anti-competitive because it prevents independent pricing and eliminates competitive dynamics.

In the context of financial data sharing schemes, data holders *will* use the schemes as a mechanism to set prices for data access. Under PSD2, the established practice has been not to charge for access to basic data. However, the Commission has acknowledged that while access to basic data is often provided free of charge, there may be justification for fees when accessing higher-quality data or additional services. [9] This reasoning could similarly extend to data access under FIDA. To address the potential anti-competitive effects of such pricing practices, FIDA requires that any compensation for data access be reasonable, transparent, and directly related to the cost of making the data available. The regulation explicitly stipulates that the compensation model must: (1) be developed using objective and transparent methodologies agreed upon by scheme members, (2) include market data collection from both data users and data holders to determine the relevant cost elements, and (3) be periodically reviewed to reflect technological progress and avoid inflated fees. Importantly, FIDA also places a cap on fees for small and medium enterprises, limiting compensation to the actual costs incurred in providing data in those cases.

It is worth noting that similar pricing provisions can be found in other data-related legislative instruments, most notably the Data Act, which introduces a horizontal framework for access to and use of data across sectors. Under Article 9 of the Data Act, data holders may request compensation for making data available, but such compensation must be reasonable and non-discriminatory, and must not exceed the costs directly related to the data-sharing transaction, plus a reasonable margin. These provisions build upon the concept of fair, reasonable and non-discriminatory (FRAND) access that has been applied and litigated in the context of the licensing of standard-essential patents for which a FRAND commitment has been issued [10]. But as commentators have noted, “the open concept of FRAND will be [in the context of the Data Act] specifiable to individual circumstances only through practice in negotiations, contracting, reasoning and litigation over time, and experience by market actors and courts.”[11] In the context of FIDA, the operational meaning of “reasonable” compensation will similarly evolve through iterative institutional and market practice, potentially requiring judicial clarification or regulatory guidance to ensure consistency and legal certainty. Thus, while the FRAND requirements, the fee cap and review mechanism are strong features of FIDA’s framework, they must be complemented by broader monitoring of technical accessibility and institutional support for smaller actors to fully level the playing field.

## 4 Do the Schemes Restrict Competition by Effect?

Even if the object of the scheme is not anti-competitive, its effects could still restrict competition in significant ways. These effects could arise because of the design of standard-setting process. Through this process, incumbents may seek to exert influence over the development of data standards, potentially steering them in ways that favor their own technologies or business models. How can this competitive risk be mitigated?

The Commission’s Guidelines provide clear recognition that the governance and openness of standard-setting processes are crucial to ensuring that collaborative initiatives do not become vehicles for foreclosure or collusion. The governance requirements under FIDA, particularly those related to representation and decision-making reflect this recognition.

For example, by requiring a fair and equal representation of both data holders and data users in the governance of financial data sharing schemes, FIDA seeks to balance the interests of all stakeholders. This provision is designed to prevent capture of the scheme’s governance by dominant incumbents who might otherwise shape standards to reflect their own technological preferences or business models. By mandating fair and equal representation in the decision-making structure, FIDA seeks to ensure that the interests of smaller firms, new entrants, and consumers are reflected in how standards are defined and applied. The requirement also supports transparency and checks on self-serving behavior by powerful actors, making it more difficult for standard-setting to become a tool for strategic exclusion.

A related competitive concern in relation to the governance design of the schemes is foreclosure of non-members from accessing data. If access to this data is limited to members of a scheme, non-members—especially smaller or independent firms—may be excluded from the market. This could create barriers to entry for new competitors, reinforcing the dominance of established players and stifling the diversity and innovation that often come from smaller firms or startups. At the same time, in EU competition law, such foreclosure generally requires a showing that the input in question represents an “essential facility”, which is not easily done in the case of data [12].

Anticipating this problem, foreclosure of non-members from accessing essential data is explicitly mitigated by FIDA’s requirement that the financial data sharing scheme remains open to new members at any time and under the same terms and conditions as existing members. This ensures that any data user can participate in the scheme if they meet the “objective criteria” defined in its membership rules. Additionally, the regulation prohibits unjustified differentiation or preferential treatment among members, reducing the possibility of exclusionary practices within the scheme itself. In theory, this should neutralize concerns about input foreclosure.

However, the effectiveness of this solution depends heavily on how the “objective criteria” for membership are defined and applied in practice. Criteria that appear neutral on their face—such as technical capacity or security standards—could still be framed or interpreted in ways that disproportionately burden smaller firms or new entrants. For instance, demanding technical interoperability standards may require significant investment in infrastructure that smaller firms cannot afford. These conditions risk creating an uneven playing field where only larger, established firms can compete effectively, potentially forcing smaller players out of the market or discouraging new entrants from participating altogether.

To summarize, financial data sharing schemes may contain elements that potentially restrict competition, either by object or by effect. Standardized fees for data access, excessive representation of data holder in the governance bodies of the schemes, or overly burdensome membership criteria could raise significant competition concerns. In the following Section, I turn to the question of the allocation of institutional responsibility for raising such concerns in the EU legal order and the criteria that the schemes will have to meet to benefit from an exemption from the application of Article 101.

## 5 Can the Schemes be Exempted From the Application of Article 101 TFEU?

Under FIDA, financial data sharing schemes must be notified to national *competent* authorities, which will assess whether their governance frameworks meet FIDA’s requirements. FIDA does not specify precisely which authorities will be designated to fulfill this oversight role, although it appears unlikely they will be the same as national *competition* authorities. This is primarily because the oversight envisioned under FIDA is regulatory rather than strictly competition-focused. Compliance with FIDA therefore reduces, but does not eliminate, the risk of infringement under Article 101(1). As is well established in existing case law of the Court of Justice of the EU, for example the *Bpost* case (C-117/20), sector-specific regulation, such as FIDA, does not provide a legal safe harbor against competition law scrutiny. FIDA states explicitly that it “does not affect the application of EU or national rules of competition of the [TFEU] and any secondary Union acts.”

Accordingly, it remains crucial to determine whether potentially restrictive elements of these schemes qualify for exemption under Article 101(3), which permits restrictive agreements only if four cumulative criteria are fulfilled: (1) the agreement contributes to improved production or distribution, or promotes technical or economic progress; (2) consumers receive a fair share of the resulting benefits; (3) the restrictions are indispensable to achieving these benefits; and (4) competition is not eliminated in a substantial part of the market. National competition authorities are responsible for assessing compliance with these criteria, and their interpretation may vary across Member States [13]. Such variation could lead to legal uncertainty for cross-border schemes, underscoring the importance of careful and consistent assessment.

In this section, I undertake such high-level assessment, drawing a functional parallel to standardization agreements [14]. Like standardization agreements, FIDA schemes involve competitors collaborating to establish common technical and procedural standards aimed at interoperability, efficiency gains, and market integration. The Commission’s established analytical framework for standardization agreements articulated in the Guidelines thus provides a valuable benchmark for evaluating FIDA’s governance structures and operational rules.

The Guidelines highlight that standardization agreements typically qualify for exemption under Article 101(3) when they are open, transparent, and grant access on FRAND terms. Illustrative examples such as GSM standards in telecommunications, USB standards for digital connectivity, and MPEG-2 standards in video compression clearly demonstrate how collaborative standard-setting can lawfully unlock innovation and deliver substantial consumer benefits. Similarly, FIDA schemes seek to overcome costly data integration barriers and facilitate fintech innovation—including personalized finance apps, sophisticated credit scoring models, and multi-provider aggregation services. Given these goals, FIDA schemes are well-positioned to fulfill the first criterion of Article 101(3), namely the promotion of technical and economic progress.

The second criterion, that consumers must receive a fair share of the resulting benefits, is also comfortably satisfied. As with technology standards, consumers benefit from financial data interoperability through broader product availability, improved comparability, and lower switching costs [15]. FIDA’s focus on customer consent, secure access, and open participation ensures that data-driven services can develop—delivering meaningful improvements in convenience, pricing, and service personalization. This aligns with the benefits identified by the Commission in its favorable assessment of, for example, MPEG-2 standardization efforts, where the agreement on a video compression was seen to significantly improve consumer experience and product functionality, without restricting downstream innovation.

However, an important structural distinction should be noted: unlike MPEG-2 case, which involved patent pools that centralized intellectual property rights through common licensing arrangements, FIDA schemes do not involve pooling or centralizing data as would be the case with a data pool as a functional equivalent of a patent pool. Instead, the schemes facilitate decentralized, bilateral exchanges of consumer-authorized data among multiple market actors.

This distinction has implications for analyzing the economic and competition effects of the schemes under the first two prongs Article 101(3) TFEU. Bilateral exchange arrangements under FIDA schemes may more readily satisfy these criteria than centralized data pools. Because their efficiencies arise primarily from interoperability, standardized infrastructure, and technical compatibility, rather than economies of scale in analytics, as is the case with data pools, the schemes are directly linked to tangible technical progress. Furthermore, since FIDA schemes prioritize consumer-authorized data transfers in consumer-facing financial services, the consumer benefit condition is more transparently met through direct enhancement of consumer agency and choice, unlike data pools, which are directed primarily at firms.

The third criterion—indispensability of restrictions—is specifically addressed in the Guidelines, which recognize restrictions as *indispensable* if they directly facilitate technical efficiency, security, balanced governance, fair participation, or cost-based fee-setting. A relevant illustration of what constitutes a *dispensable* restriction comes from the Commission’s Philips/VCR decision, which rejected a “grant-back” clause requiring licensees to transfer improvements or innovations back to the original patent holders. The Commission found this obligation unjustifiably restrictive, as it limited independent innovation without being necessary to achieve the licensing arrangement’s objectives.

In the context of FIDA schemes, a comparable restriction might involve mandating data users—such as fintech firms or third-party providers—to share insights, analytical tools, or innovations derived from accessed data with incumbent data holders. For example, a rule requiring fintech companies to disclose proprietary credit-scoring algorithms or customer analytics to incumbent banks as a condition for accessing customer financial data would similarly be regarded as unnecessarily restrictive and likely fail the indispensability criterion.

Finally, the fourth criterion—that the agreement must not eliminate competition in a substantial part of the market—is generally satisfied in standardization agreements when participation is open, non-exclusive, and conducive to independent innovation. In decisions involving MPEG-2, the European Commission explicitly recognized that competition remained robust because firms retained the freedom to innovate in implementation, product features, and complementary services, despite adhering to common standards.

Financial data sharing schemes under FIDA comfortably meet this fourth criterion due to their explicit requirements for open participation, non-discrimination, and fair access as well as decentralized implementation, which creates opportunities for innovation. Still, while such decentralization can promote flexibility and innovation, it also creates a heightened risk of inconsistent technical implementation compared to centralized patent or data pools, where uniformity of standards is intrinsic. This difference underscores a potential weakness in FIDA’s approach: the absence of clearly prescribed substantive standards could lead to fragmented interoperability and technical barriers similar to those experienced under PSD2. It is this crucial risk—and its implications for competition and innovation—that the following Section addresses.

## 6 Other Competition Risks Posed by the Schemes

As the preceding analysis demonstrates, the governance requirements for financial data sharing schemes under FIDA have been explicitly designed to reflect core competition law principles. By mandating open and non-discriminatory membership criteria, transparent governance processes, fair representation of data holders and data users, and periodic review of compensation models and technical standards, FIDA seeks to prevent exclusionary practices, collusion, and the creation of artificial barriers to market entry. Additionally, the inclusion of consumer organizations within governance structures further aligns the schemes with competition law objectives, aiming to safeguard consumer interests and promote innovation.

Nevertheless, while these governance safeguards provide important protections, they do not fully ensure that the substantive standards developed by scheme participants will be free from anti-competitive effects. As discussed earlier, standards can limit competition when they are excessively complex, requiring infrastructure investments beyond the capacity of smaller firms. This was a concern, for example, in an investigation launched by the Commission into the standardization process for payments over the internet undertaken by the European Payments Council (EPC) as the coordination and decision-making body of the European banking industry for payments. In the course of the investigation the EPC announced its decision to stop the development of some of its standardization initiatives. As a result, the complainant in this case, Sofort AG, withdrew its complaint. Under these circumstances, in 2013 the Commission has decided to close its investigation.

Standards can also be anti-competitive when they are inadequately defined or underdeveloped, a risk clearly illustrated by the experience under PSD2, which required banks to grant third-party providers (TPPs) open access to payment account data via APIs. Yet the European Banking Authority's Regulatory Technical Standards RTS, although intended to harmonize technical interfaces, stopped short of mandating uniform API specifications. Consequently, banks often introduced incomplete or restrictive APIs, citing security or technical concerns as justification. A 2021 European Commission study on PSD2’s impact confirmed that this vagueness led to considerable interpretative differences between banks and TPPs regarding accessible data and the conditions for data access, allowing some banks to engage in data “gatekeeping” practices [3, p. 144].

In practice, the resulting underdeveloped and inconsistent API standards created artificial hurdles for smaller fintech providers, including limited API functionality, performance issues like high latency, and unnecessarily complicated authentication procedures [3, p. 128]. Rather than fostering competition and innovation, these inadequacies effectively shielded incumbents, suppressed new service development, reduced consumer choice, and hindered market entry. This outcome demonstrates that insufficiently precise standards can significantly compromise competition, including thorough practices such as intentional degradation or corruption of the quality of shared data sometimes referred to as data poisoning. Because such practices typically follow from unilateral action rather than collective conduct, they are unlikely to constitute a restriction of competition by effect under Article 101 and must be addressed at the level of the standard itself.

In the UK, the issue of inconsistent API standards and access was tackled through the establishment of the Open Banking Implementation Entity (OBIE), a centralized body specifically tasked with implementing and overseeing open banking under the UK’s Competition and Markets Authority (CMA) remedies. OBIE was responsible for developing a single, standardized set of technical requirements for APIs, ensuring consistency in implementation across all banks. This avoided the fragmented approach observed under PSD2 in the EU. The CMA mandated the nine largest banks to comply with OBIE’s open banking standards and ensured their APIs met the required functionality, performance, and security levels. Compliance was monitored centrally by OBIE and the CMA. OBIE provided rigorous oversight, testing APIs for quality, availability, and compliance. Any non-compliance or barriers introduced by banks (e.g., technical restrictions or delays) were promptly identified and addressed. OBIE also acted as an intermediary, helping TPPs resolve issues with access or compliance. This proactive approach ensured smaller players could participate without facing undue barriers. By creating a single entity to set standards, enforce compliance, and resolve disputes, the UK avoided the inconsistency and enforcement challenges observed in the EU [16]. Open banking in the UK has been widely regarded as a success, with APIs functioning reliably and fostering innovation.

In the PSD2 context, API-standardization was seen as antithetical to the goal of fostering competition [17]. Allowing banks to develop their APIs within a set of general guidelines provided flexibility for innovation and differentiation, or at least that was the official position. But the position is seriously flawed and, to my knowledge, has not been challenged in the academic literature. While the intention was to allow banks flexibility for innovation and differentiation, in practice, this approach created fragmentation, inconsistency, and barriers to entry, undermining the very competition it sought to promote. By allowing banks to develop their own APIs within a broad set of general guidelines, the EU failed to ensure interoperability across financial institutions. As a result, TPP such as fintech firms, faced significant operational challenges integrating with multiple banks, each of which implemented APIs differently. This lack of uniformity disproportionately harmed smaller players, who lacked the resources to navigate bespoke systems, while favoring incumbents that could exploit their market position [18].

The reasoning that strict standardization would hinder competition appears, in retrospect, to be a convenient cover-up for political reluctance on the part of the Commission to interfere with national banking interests. Banks in various Member States often wield significant political and economic influence, which may have contributed to a preference for a decentralized, less prescriptive regulatory framework. By refraining from imposing harmonized standards, the Commission avoided direct confrontation with powerful banking lobbies at the national level. However, this compromise came at the cost of market efficiency, innovation, and consumer welfare, as the fragmented approach undermined the goals of PSD2.

Moreover, the argument that innovation requires flexibility and fragmentation is misguided. A clear, harmonized API standard, as seen in the UK’s Open Banking framework, does not preclude differentiation or innovation. Instead, it provides a robust technical foundation on which participants can innovate and compete. Standardized APIs ensure interoperability and fair access while still allowing firms to develop value-added services, enhance user experience, and introduce unique features that benefit consumers. Without this baseline infrastructure, the fragmented landscape in the EU has created inefficiencies, higher costs for TPPs, and ultimately fewer choices for consumers. In the context of FIDA, a similar risk exists: without clear, harmonized guidelines and strong EU-level oversight, financial data sharing schemes could exploit these gaps to limit competition, especially across multiple jurisdictions. As I have argued elsewhere, the failure to provide guidelines, for example in respect of data quality, undermines the promise of using the financial data space to promote the development of AI applications [15].

There is a possibility that a harmonized approach to financial data sharing standards will ultimately be adopted under FIDA, as Article 11 provides a mechanism for the European Commission to intervene when necessary. Specifically, if no financial data sharing scheme is developed for one or more categories of customer data listed in Article 2(1) and there is no realistic prospect of such a scheme being set up within a reasonable timeframe, the Commission is empowered to adopt a delegated act in accordance with Article 30. This delegated act would supplement FIDA by specifying clear modalities under which data holders must make customer data available pursuant to Article 5(1). The provisions would include: (a) common standards for the data and, where appropriate, the technical interfaces to enable customers to request data sharing, (b) a model to determine the maximum compensation that a data holder is entitled to charge for providing access, and (c) the liability framework for entities involved in making customer data available.

This empowerment of the Commission represents a safeguard to prevent the fragmentation and inconsistencies seen under PSD2. By granting the Commission the authority to establish common standards and frameworks, FIDA recognizes the risk that, without intervention, the absence of a scheme for certain categories of customer data could lead to inefficiencies, unfair access conditions, and anti-competitive practices. If the delegated act is triggered, the Commission would be able to mandate harmonized technical standards and compensation models, addressing concerns about interoperability, excessive fees, and liability. This approach would ensure that financial data sharing schemes remain aligned with competition law principles, providing a consistent and fair infrastructure for all participants across the EU while avoiding the pitfalls of fragmentation observed under PSD2.

## 7 Conclusion

The governance framework for financial data sharing schemes under FIDA represents a careful effort to integrate EU competition law principles—particularly those embodied in Article 101 TFEU—with sector-specific goals of interoperability, innovation, and consumer empowerment. FIDA’s provisions, including open membership, non-discriminatory access conditions, and transparent governance processes, significantly reduce the risks of anti-competitive behavior. Nevertheless, the absence of directly mandated substantive technical standards leaves significant gaps that may replicate interoperability issues previously experienced under PSD2, undermining the competitive potential of these schemes.

While a direct public intervention to address these gaps remains an option, future research should explore how insights from the literature on transnational private regulation might enhance the effectiveness of the schemes contemplated by FIDA. This body of research has demonstrated how private contractual and organizational mechanisms, not dissimilar to those represented by the schemes, can foster the development of standards that are effective in achieving their intended objectives whilst being perceived as legitimate by stakeholders [19]. Additionally, comparative analyses of similar regulatory initiatives, such as open finance programs in the UK and Brazil, could further inform the optimal balance between flexibility and regulatory certainty. By building on these foundations, future scholarship could provide valuable guidance for strengthening not only FIDA but also broader European initiatives in sectoral data spaces and open data governance.

## Ethics Statement

The author has read and follows the ethical requirements for publication in Data in Brief and confirming that the current work does not involve human subjects, animal experiments, or any data collected from social media platforms.

## CRediT Author Statement

**M. Konrad Borowicz:** Conceptualization, Methodology, Writing – original draft, Writing – review & editing, Project administration, Funding acquisition.

## Declaration of Competing Interest

The author declares no competing financial or personal interests that could have influenced the work reported in this manuscript.
